# The Use of Spatial Video to Map Dynamic and Challenging Environments: A Case Study of Cholera Risk in the Mujoga Relief Camp, D.R.C.

**DOI:** 10.3390/tropicalmed7100257

**Published:** 2022-09-22

**Authors:** Andrew J. Curtis, Felicien Maisha, Jayakrishnan Ajayakumar, Sandra Bempah, Afsar Ali, J. Glenn Morris

**Affiliations:** 1 Department of Population and Quantitative Health Sciences, School of Medicine, Case Western Reserve University, Cleveland, OH 44106, USA; 2 Emerging Pathogens Institute, University of Florida, Gainesville, FL 32601, USA; 3 Department of Geography, Kent State University, Kent, OH 44242, USA; 4 Department of Environmental & Global Health, College of Public Health and Health Professions, University of Florida, Gainesville, FL 32601, USA; 5 College of Medicine, University of Florida, Gainesville, FL 32601, USA

**Keywords:** GIS, spatial video, mapping, relief camp, cholera

## Abstract

In this paper, we provide an overview of how spatial video data collection enriched with contextual mapping can be used as a universal tool to investigate sub-neighborhood scale health risks, including cholera, in challenging environments. To illustrate the method’s flexibility, we consider the life cycle of the Mujoga relief camp set up after the Nyiragongo volcanic eruption in the Democratic Republic of Congo on 22 May 2021. More specifically we investigate how these methods have captured the deteriorating conditions in a camp which is also experiencing lab-confirmed cholera cases. Spatial video data are collected every month from June 2021 to March 2022. These coordinate-tagged images are used to make monthly camp maps, which are then returned to the field teams for added contextual insights. At the same time, a zoom-based geonarrative is used to discuss the camp’s changes, including the cessation of free water supplies and the visible deterioration of toilet facilities. The paper concludes by highlighting the next data science advances to be made with SV mapping, including machine learning to automatically identify and map risks, and how these are already being applied in Mujoga.

## 1. Introduction

Temporary or unofficial living environments, for example, some slums and informal settlements (SIS), pose some of the greatest health challenges [1,2,3,4,5]. From a spatial science perspective, these environments also suffer from little official data, while also lacking the resources needed to acquire new data [6,7], especially at a more granular scale. In this paper, we draw on previous mapping experiences in different SIS environments to consider the life cycle of a relief camp set up after the Nyiragongo volcanic eruption in the Democratic Republic of Congo on 22 May 2021. More specifically we investigate how the deteriorating condition of the camp has led to an increased localized cholera risk.

### 1.1. Spatial Data Collection in Challenging Environments

Mapping contextualized granular space is important to investigate local disease risk and know where to target intervention. Topics such as understanding daily activity spaces; how the journey from a water point to a residence adds risk; how proximity to a trash dump is linked to disease; the interconnection between human activity and fecal contamination of drainage channels [8] or mosquito breeding all require granular spatial data. Further complexity is added when we consider changes across time, especially as SIS environments tend to be highly dynamic in nature [9]. Different methods to address this data gap have included the use of high-resolution imagery [10,11], UAV flights [12], on-the-ground surveys [7], participatory mapping [13], web-based crowdsourcing [14] and open source data mining [15,16]. While display and access to the new spatial data can include platforms such as OpenStreetMap [17], for many locations issues still remain regarding the replicability, transferability, and sustainability of a reliable local mapping solution, especially one that can be updated and imbued with appropriate context [18].

More specifically, when considering granular scale cholera risk, maps should include case locations (if known), domicile patterns, Water, Sanitation and Hygiene (WASH) infrastructure, and related environmental factors such as drainage patterns and natural water sources. Ideally, these spatial layers should be contextualized with local insights to interpret the nature and severity of risk. For example, not all drainage channels pose the same risk to those living close by, while even geographic variation along the same channel can vary depending on local physical relief, behaviors, and politics.

While the authors of this paper have developed mapping approaches to consider health problems in different SISs, an arguably even more problematic situation can arise in temporary relief camps. These data-poor, highly dynamic, densely packed settlements have all the potential conditions in which disease outbreaks might occur, especially enteric diseases such as cholera [19,20,21]. While these camps may have an initial advantage of government or NGO funding and oversight, including building access to WASH features, the on-the-ground situation varies between camps and can change over time. Soon the same questions posed in an SIS will arise; where are there environmental risks such as standing water or mud, especially after rains [22]? Where does trash accumulation pose health risks, such as water pooling suitable for mosquito breeding [23]? How has the safety of the WASH features changed over time [24]? More specifically is the drinking water safe, or does it fluctuate under certain environmental or meteorological conditions? To be able to answer these types of questions in a settlement that has literally just developed requires a transferable robust method specializing in local contextualized mapping. Spatial video mapping is one such method.

### 1.2. Spatial Video Field Mapping

Spatial video (SV) media are linked to geographic coordinates, often from a simultaneously collected global positioning system (GPS) receiver data stream [25]. First, field uses of the technique described here were developed in response to highly dynamic and data-poor post-disaster environments, including Hurricane Katrina, various tornadoes, and wildfire events [26,27]. These initial systems involved bulky camcorders and large external GPS receivers, so the typical mode of transport was a sports utility vehicle [28,29]. Each coordinate was added to the video frame as an electronic beep output from the GPS receiver [27]. As technology improved so the method evolved to be more flexible and suitable for any environment, and any means of transportation. The technical advances included off-the-shelf and relatively inexpensive extreme sports cameras, and bespoke software designed to maximize collaborative involvement. The first uses of this new form of SV in Haiti were on cars with cameras capturing left, right and front views, with the GPS coordinate being recorded onto the video frame as a header [30,31]. These wide-angle high-resolution cameras could record the ground by the side of the vehicle, capturing standing water or trash [31,32], while at the same time having enough of a panoramic view to place the frame within a broader context, such as nearby buildings, vendors, and even human activity. The small size of the camera, and the obvious downward angle of the lens, also helped in lessening any local questions about why and what was being collected. Even so, a concise explanation was prepared and available for any community asking why their neighborhood was being video mapped. The resulting image, after downloading and displayed in the software supplied with the camera, became a digitizing source to create maps of local risks, such as the local drainage pattern, standing water and mud, trash accumulation, presence of animals, and different human activities such as children playing in unsafe environments. This type of car-based SV collection also proved to be an excellent tool for collaboration, with projects developing in Cambodia, Colombia, Ghana, Kenya, Malawi, Nicaragua and South Africa. It soon became apparent that in many of these environments the narrow passages were unsuitable for automobiles, so other modes of transport including bikes and boats (for flooded areas), and especially walking collections were utilized. Different spatial sampling designs were employed; where possible, automobiles would capture a more comprehensive neighborhood coverage and even multiple areas for comparison. For walking collections [33], transects were used to capture spatial slices of the SIS, and then on return visits at different time intervals allowing for comparative change mapping [34,35]. Topical investigations included mapping specific health risks associated with cholera, dengue or malaria (Haiti, Colombia, Ghana) [23,36], the risk of flooding over time (Bangladesh, Cambodia, Kenya) [34], the impact of trash and living close to large dumps (Cambodia), and following activity paths, such as the water carrying route from well to residence (Haiti and Tanzania) [33].

SVG also started to be incorporated into epidemiological field studies. In Haiti for example, teams would collect monthly water samples from within the SIS while simultaneously recording the microenvironments around each test site [24]. This allowed for later mapping and analysis to identify proximate environmental risks, such as the spatial interrelationship between a well and a local drain, or how buckets standing on muddy ground and then lowered into the reservoir could taint the supply, or even how the physical nature of the waterpoint changed over time, such as the concrete degrading and being replaced by an enclosed plinth [37]. These surveys provided visible (and therefore mappable) context to the epidemiological data being collected.

Technology continued to improve with the sports cameras now being replaced by cheaper, more rugged body cameras typically used by the police. These cameras also had a larger data storage capacity, better battery life, better audio recording and automatic low light settings. At the same time, new software was developed by the team to enhance visualization and add new functionality such as being able to digitize directly onto the map that showed both the video frame and its associated coordinate. Further advances included being able to repair the GPS path, this being especially important as the metallic buildings, overhanging roofs, narrow corridors, covered cameras due to security concerns, and even the latitude could lead to considerable route error. The addition of the video meant that these paths could be corrected manually for mapping purposes [33], but then, using the new software, these new GPS tracks could again be synced with the video [35]. This same approach could even be used to create a new track for videos where no GPS had been collected.

A key SV development goal was to have local collaborators participate in as much of the research/work as possible, not just in data collection but also in collaborative discussions, mapping and analysis. To this end, all software development was designed to keep technical requirements to a minimum. One of the most important advances was improving how video data could be shared. The first video files were recorded in High Definition as this provided the best digitizing source. The resulting file size was problematically large for uploads, especially given local internet access challenges. The only solution was to hand carry data whenever a member of the team traveled as local mailing services were often not an option. To solve this problem secure compression software was developed. As approximately 20 frames were collected for each second of video, reducing the number of frames uploaded reduced the file size without noticeably affecting the image quality as a source for digitizing. Other features in the software added further utility, such as being able to extract audio from the video, an important feature for the next advance in SV data collection, which was geonarratives [38]. As with other software developed by the team, all are freely available. This means that the only logistical challenges in terms of costs for SV deployment are the camera cost (approximately $170 at the time of writing), any costs for local data collection, and how to deliver the camera to the site. In all previous projects, the cameras have been bought by the authors of this paper, sometimes using grant funding, and then hand-carried by team members traveling to the study location. On-the-ground data collectors are already being paid to collect epidemiological data (as in the example described in this paper), or if the request for collaboration originated from the location being mapped, there was already local capacity. Obviously, every setting is different, and the biggest commonly faced challenge is the transfer of the cameras and then data given the vagaries of local postal delivery. However, so far, no situation has proven too difficult to navigate.

While SV mapping data enrich our understanding of SIS-type environments, adding in a simultaneously collected commentary dramatically improves the final map by adding local context. Instead of being able to see, and then map, the local drainage channel, a local or expert voice can tell how the trash being dumped is the cause of local flooding [22], or how there are still mosquito clouds in the dry season because of the trapped standing water, or why residents distrust the local hospital [34]. In Colombia, these geonarratives helped explain localized patterns of dengue [36], and also the connected issue of violence [39]. In Cambodia, flooding in the informal settlements was described in terms of the individual impact it would have as the feces-tainted water would rise within the home during the rainy season. These Spatial Video Geonarratives (SVGs) might be collected as part of an SV survey, or as a supplement to ongoing epidemiological data collection. Most recently, as a result of COVID-19, Zoom based geonarratives (SVGZ) have been developed and these have proved useful in letting the team return to the original environment at a later period to discuss change. In Haiti, for example, SVGZ has been used to discuss how the field sites have changed in areas where gang violence now prohibits data collection. In order to fully leverage both field and zoom based spatially tagged text, new software has been developed, the most important of which is *Wordmapper* [40]. In *Wordmapper*, the video and transcribed text file of the interview are input and merged using their time stamp. This allows for every comment to be mapped out. By adding in keyword searches, certain comments can be highlighted both in the text bar and on the map. A word cloud helps identify co-occurring words that can further tighten the search for key terms. Themes can be added, and comments that are explicitly spatial, meaning an actual location is being described, can be marked so that output map files can be viewed on Google Earth, or further enhanced cartographically in a GIS.

With these advances, SVG projects were now generating multiple different media types, often covering several time periods. In addition, new data layers were being created through digitizing risk. To visualize and search for patterns across all these inputs required new software [41,42]. Changes were also needed in the conceptual approach of how to fully leverage the emotional content of the narratives, drawing on non-linear investigative strategies more typically applied in the spatial humanities [43,44,45]. Simply put each data/media input could help develop the exploratory question; the contextual insight could inform an idea, further verified on the new map, and in the video frame, before linking back to the text for other connected comments, maybe recorded elsewhere on the route [44].

Even the more typical epidemiological data required a different form of spatial analysis. Drawing on the team’s data science background, and the way dashboards had developed during the geospatial response to COVID-19, software was developed that allowed for visually interactive ways to explore spatial and numerical associations [37]. In this way, fluctuations around one water point in terms of the fecal coliform load could be interactively compared against all other water points for that data collection time period, or from the life history of that particular testing site, or as part of a localized pattern of potentially interconnected features [37]. By adding in the digitized environmental risk scores, and even images of each site, potential causation could also be explored from the revealed patterns.

Another data science advance explored the use of machine learning to automatically identify risk features from the SV imagery. The typical creation of localized risk maps from SV sources is manually intensive. The recent advancements in neural networks, especially convolutional neural networks (CNN), have provided an impetus for image analysis tasks such as image classification, object detection, and image captioning. Recent work using SV data in Haiti [46] had shown that an object detection algorithm based on YOLO (you only look once) could identify risk features, such as trash, standing water or even tires, buckets, drains, etc. While the model was able to successfully identify many of these features, the lack of available training data, one that could capture the regional nuances of the general categories such as the type of trash found locally, and which is quintessential for neural network models, remains one of the biggest challenges. To address this, new software was developed to allow for the easy capture of SV images to be used for model training [46]. The next generation of these models will explore how to use the connected GPS coordinate to create *automatic* mapping solutions.

Many of these advances, especially temporal comparison mapping using an SV and adding context through an SVGZ interview, will now be illustrated in terms of a near-real-time response to a post-disaster relief camp, an environment that did not exist in April 2021, and by January 2022 was already presenting localized cholera risks.

## 2. Methods and Materials

### 2.1. The Study

On 22 May 2021, the Nyiragongo volcano erupted on the eastern border of DRC and Rwanda in the Virunga National Park, resulting in approximately 400,000 being displaced. This led to a substantial relief response with NGOs providing shelter, food and associated WASH facilities [47]. The Mujoga relief camp, north of Goma City, initially housed approximately 900 households and 6300 people, though these numbers fluctuated each month due to the highly dynamic situation, especially as migrants not connected to the original eruption began to make their way to the camp. The original location for the camp was based around a few established buildings already serving the surrounding villages.

In these types of densely settled, highly fluid environments, the potential for infectious disease outbreaks is well known [48]. As is typical for many similar camps, there was no (at least to the teams’ knowledge) mapping of the camp and its surrounds. Adding to this concern was the fact that the Goma area consistently produces high numbers of cholera cases. For example, in the North Kivu health division, which is part of the general Goma area, and which specifically covers the camp, there were 15,050 cases and 135 deaths in 2017, 2967 cases and 32 deaths in 2018, 7034 cases and 52 deaths in 2019, 4068 cases and 19 deaths in 2000 and 1241 cases and 9 deaths in 2021. As a result of this area’s susceptibility to cholera, an epidemiological team already working in the Goma area repositioned resources to monitor the physical and health-related changes in the camp using SV, especially as there was no early warning system already in place. The team’s initial interest was in the relocated families who had been enrolled in part of an ongoing cholera study and whose homes had been lost or threatened by the lava flows. It soon became apparent that the new relief camp also posed a potential risk for cholera. The two questions being asked were, how was the camp changing every month, and were there developing factors that might lead to an increased disease risk, especially cholera? To answer these questions the same mapping techniques already being employed by the team in Goma, and in Haiti were used [24,31].

#### Field Data Collection, Data Transfer and Contextualization

Each month a team member would walk around the camp recording the environment with a small high-resolution body-worn video camera. This camera is discreet, rugged, and can be hand carried or fixed to a belt. It also has an inbuilt GPS receiver. The field teams had instructions to record all important features in the camp, such as tent locations, water access, toilets, etc. Data were then to be transferred back to the United States using secure bespoke compression software developed by the team. The plan was to map the changes in the camp every month.

**Figure 1:**
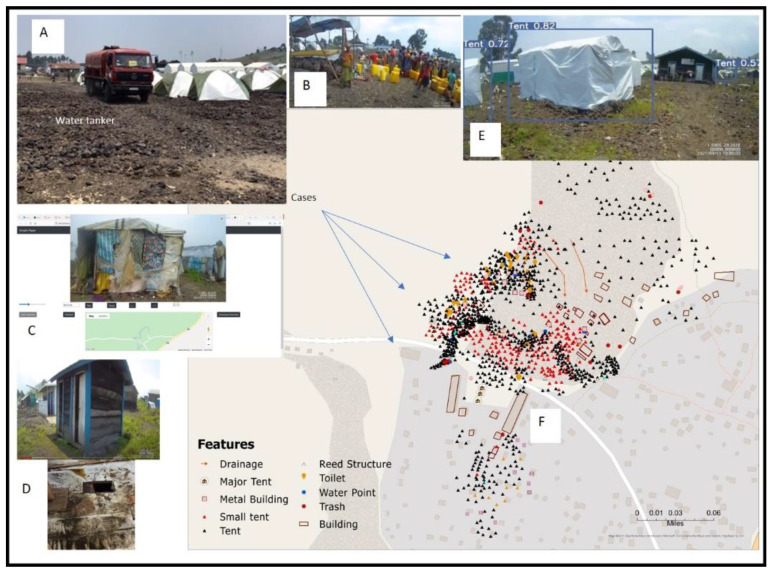
The Final Camp Map. Decisions were made to distinguish three hierarchies of tents, with small tents being the typical family abode. Other features mapped included pre-camp buildings, toilets, water access points, slope of the land and any visible risk, such as trash. The map also displays a generalized location of laboratory-confirmed cholera cases. These locations would be marked as an X on the field maps returned by the DRC team, though the exact tents are not shown to preserve confidentiality. (**A**) Tanker delivering water to the camp. (**B**) People waiting for the water delivery at the main access point. (**C**) An example of an informally constructed tent displayed within the Spatial Video Geonarrative Zoom software. (**D**) Examples of the camp toilets; the outer toilet structure and inside a feces-stained hole. (**E**). An example of an official tent for the camp displayed in the bespoke machine learning software developed by the team. (**F**) The composite map of the camp that evolved through adding each month’s spatial video.

These monthly maps would display key locations such as tents, toilets, and water sources (Figure 1) using similar cartographic approaches developed in Ghana, Haiti, and Kenya [22,23,34,35]. In addition, other environmental features pertinent to enteric diseases, such as drainage and slope were also to be digitized. Every month a feedback process with the field team would be used to verify mapped objects and help contextualize camp functions. These interactions would include a monthly zoom meeting, email exchanges with overview summaries, responses to questions, and digital maps sent back to DRC to be edited or written on. A zoom-based spatial video geonarrative (SVGZ) was also used to conduct a virtual walkthrough of the camp. For the SVGZ the field team would be asked questions while both groups watched the video, stopping at different sections of the camp to discuss specific health risks. Bespoke software working in connection with the Zoom transcription (Figure 1C) would be used to match the original coordinate of each video frame to the associated text of the conversation ([40]). This spatially encoded text was then input into *Wordmapper* for contextual mapping as it would be during a normal SVG. Output from the SVGZ would be used to enrich the original maps through object clarification (for example how a building should be labeled), and additional context such as explaining any changes that were being seen in the camp.

## 3. Results

SV data were collected every month from June 2011 to March 2022. The time spent on each SV collection varied depending on how much area was covered, and how long the team stopped at key locations, such as the main water distribution center, or the toilets, to capture local detail. After each collection, the video was downloaded from the cameras and sent to the US team using the bespoke compression software. The specifications of the compression, including how much the image quality had to be reduced to speed transfer, evolved after the first few months so that certain features could be better discerned for mapping. After each SV collection, the main camp was mapped and compared to previous iterations to identify change. Initially, two different map makers used the video, one mapping directly in CameraPlayer, a bespoke software developed to display the video along with a moving map cursor, and the other map maker using Google Earth and ArcMap GIS. These map-making choices were individual, though the finished product was then compared, and a single map style, with consistent map object symbolization, was chosen for the remainder of the monthly maps. This same map style was used in the final master map which is shown in Figure 1 to aid subsequent comparisons.

The original location of the camp grew around several already existing buildings (Figure 1F) which had previously served a service role for the surrounding communities. Some of these, such as the church and school, continued in that function even though the number of people had grown substantially. Indeed, some of the larger tents were designed to expand the capacity of these functions to cope with the influx of displaced families.

**Figure 2:**
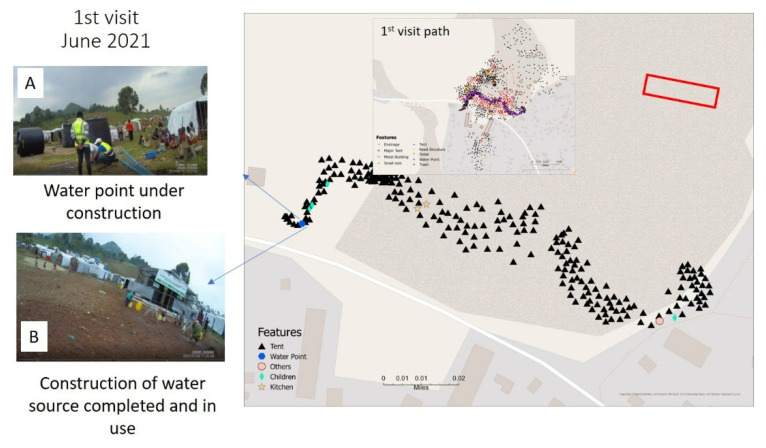
The first (June 2021) SV map for Camp Mujoga. The construction of the main water distribution center is shown in image (**A**). The second image, (**B**), is taken from the July SV collection and shows the completed water center. This location also corresponds to Figure 1B, while Figure 1A shows the water truck arriving to fill the reservoir. The inset map in Figure 2 is a version of the final map (Figure 1) with the June SV path overlaid in purple.

On the first map made after the June SV, many of the central features of the camp were initial identified, some of which had only just begun to be constructed (Figure 2).

**Figure 3:**
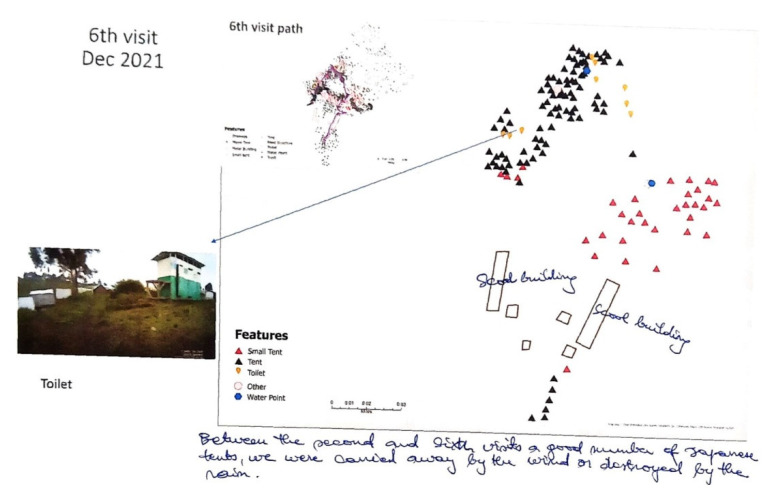
The December 2021 SV Map with Additional Field Team Notation. The field team has identified the role of the main buildings. Moreover, in response to the question as to why some of the tent areas had shifted in the camp, a hand-written explanation is offered below the map. This note says that one type of official relief tent did not perform well under local meteorological conditions, especially in high winds, and these had to be replaced.

After each month, questions were asked of the field team to help verify and label the cartography. One approach used was to send a draft map to the field team as a PDF, while a Google Earth KMZ file was also available to allow for more detailed zoomed-in digital looks. The PDF maps would be returned with objects marked on, and with additional written descriptions to help further improve the cartography. In some instances, this feedback helped identify the role of features, and sometimes it provided an explanation for changes between the months (Figure 3).

As illustrated in Figure 3, the number, placement, and type of tent construction changed with each month’s SV collection. The original displaced families would live in one of three different “official” tent designs, two of which can be seen in Figure 1 (Figure 1A,E). Across the months the distribution of these tents changed, some areas thinned as families returned home, while others were relocated because of wind, rain and flooding (as was described in Figure 3). As there was no organization to the tent locations, some of the groups of tents also realigned as friends, families and neighbors coalesced based on culture or familiarity. There appeared to be no camp oversight guiding this process with these relocation decisions being spontaneous. Other tent movements occurred because of families wanting to be closer to WASH features or wishing to move away from less desirable environments such as being downwind of the toilets.

**Figure 4:**
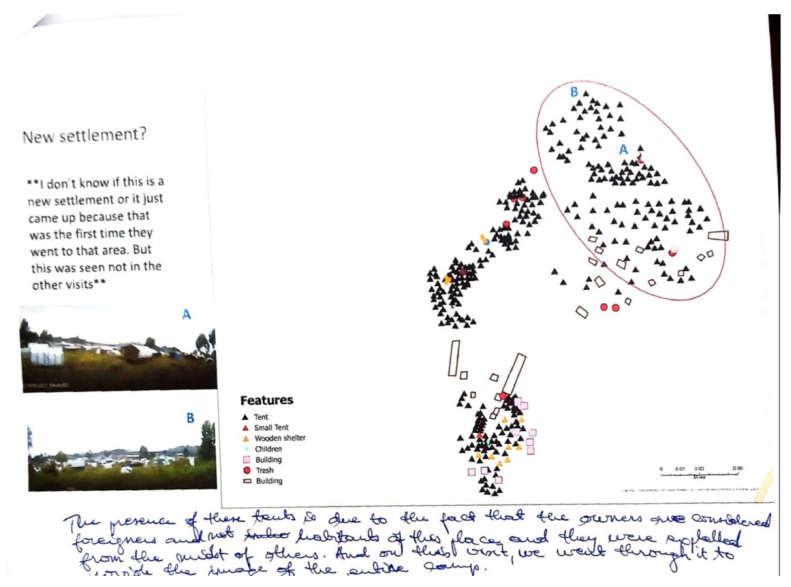
SV Map Showing Major Camp Realignment. One month’s SV saw a whole section of tents disappearing, and then others emerging. In the map, an area of new tents has been circled in red. When questioned about this the field team added explanatory handwritten comments that this had been the result of an internal camp conflict with “foreigners”, meaning later settlers in the camp, being expelled to the outer fringe by the original refugees. The inset images of A and B, which are also noted on the map, provided additional context for the field team to comment on.

Another visual indicator of the cause of this conflict, and in general the influx of people not originally displaced by the volcano, was the emergence of informal tents, homes constructed of whatever material was available, including plastic sheets, sticks, etc. (Figure 1C, the main image in the previously described SVGZ software being an example). Inhabitants of these “informal” tents included families from the region living in poverty who believed that the camp would provide free and safe services, including clean water. Other unofficial residents were those originally reliant on the displaced families for employment, and even though they had lost their homes and income, they did not directly qualify for relief assistance. This influx of “others” also led to a further redistribution of tents as some of the incoming groups were forced to the northeast (Figure 4). 

The SV also captured some of the contextual behaviors in the camp. Communal cooking facilities, food distribution, and particularly relevant to enteric disease spread, the environments and activities around water access points and toilets. One of the main water distribution points has already been described, but there were several other smaller plastic barrels on wooden frames, often situated close to toilets. The field team believed these were primarily supposed to be for household cleaning and not drinking, though, and as found in other settings, the concern was that camp children might still drink from these sources. Indeed, as will be reported in the discussion section, most of those reporting cholera-like symptoms in the camp were children.

There were also multiple toilets around the camp, built of different materials, usually as a block of 2 to 4 stalls with a privacy shield (wooden or metal) around a hole in the ground (Figure 1D). Once the hole was filled then the structure would be abandoned. On many of the SV trips, the camera was used to assess the cleanliness of the toilets, which varied considerably. This situation changed for the worse at the beginning of 2022 when the original NGO funding period ended. As a result, there was no longer any free water access, and the toilets were no longer being cleaned. The water distribution center shown in Fig 1a and 1b had gone and the smaller water containers dotted around the camp had also been removed. To discuss this potentially harm-causing change of events, both US and DRC teams conducted a spatial video zoom geonarrative. The DRC team described how the toilets were no longer being cleaned by the NGO, so sometimes a local gathering of tent families claimed ownership, took on the duties of cleaning, but then restricted use to their immediate community. When the combined teams watched the latest video, conditions had visibly deteriorated, with feces marks evident on the floor and walls of the toilets (Figure 1D).

A further change was that there was no longer free food being distributed. The point was raised that many of the tent dwellers would struggle to pay for such basic needs, not just food but also charcoal or wood needed for cooking and heating. While during the day the tents were too hot for children (many could be seen sleeping on the ground around their tents) at night it was cool enough that families needed a heating source.

Another topic raised was safety in the camp. As seen in many SIS environments, camp security at night was always a problem, especially for women. The lack of lighting and little official oversight (probably getting worse after the funding ceased) meant that night visits to the toilet were deemed too dangerous, with people resorting instead to “flying” toilets, which means defecating into bags and then tossing them from, and around the tent.

In summary, by the last SVG collection mapped for this paper, access to clean water, a deterioration in sanitation, questionable food availability and safety issues all made the camp vulnerable to the potential of an enteric disease outbreak.

## 4. Discussion

The Nyiragongo volcanic eruption of 22 May resulted in a large displacement of families, some of whom moved to the Mujoga relief camp north of Goma. As in many post-disaster settings, an initial concern was how to prevent and control any infectious disease outbreaks in a stressed, densely packed population who were now mainly living in tents, and who had to rely on unfamiliar food, water distribution, and toilet facilities. In these settings, a major concern is fecal-oral disease transmission, especially cholera. Typically, in these circumstances little if any mapping is available to ascertain where risk is greatest, how that risk changes, and where suspected disease cases are located. For many such camps, there is little opportunity to develop data-driven interventions including the opportunity to visualize dynamic changes in the camp. In this paper, we first provided an overview of a mapping technique, spatial video, which is suitable for such environments. We described how SV and additional contextualization has previously been used to map cholera risk in SIS settings. We then applied these same techniques to the relief camp with monthly SV visits providing a mapping source enriched with contextualization through written comments, notated maps, and SVGZ. The result of this work was the creation of physical and digital cartography that can be updated on a regular basis, that can be used to assess localized risk, and on which future spatial epidemiological investigations can be performed.

**Figure 5:**
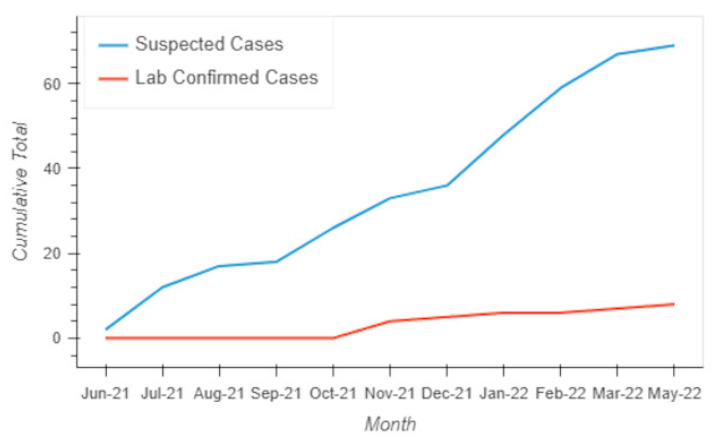
Cumulative Suspected and Confirmed Cholera cases in the Mujoga Camp.

The monthly mapping of Mujoga revealed how camp conditions had deteriorated since the beginning of 2022 which in turn increases the likelihood of future enteric disease outbreaks. Adding concern to this situation was that there had already been 68 people who had displayed cholera-like symptoms. These are likely to be an undercount due to there being no official alert or response system. The only process in place was that samples from suspicious cases were collected by a UNICEF-funded Congolese Red Cross and then passed on for laboratory confirmation, the results of which can be seen in Figure 5.

The first suspected cases were detected in June when two 11-year-old girls from Kanyarutshinya were suspected of having cholera, though neither was confirmed through laboratory analysis. The highest rise in laboratory-confirmed cases occurred in November with 4. Another confirmed case occurred in December and then January. Most of those reporting cholera-like symptoms were reported just before or after the major change in the camp’s funding when safe water deliveries stopped, and the toilet conditions deteriorated. While some of the original displaced camp population had returned home by this point, their numbers had been replaced and even exceeded by others drawn to the camp by the initial promise of free services. By early 2022, the lack of freely available clean water and food, unclean or territorially protected toilets, safety issues at night, and no within-camp health or medical services, meant that camp conditions had declined to a degree warranting concern.

In many ways, the current camp conditions mirror other SIS neighborhoods. Possibly, as has been seen in Haiti, this type of post-disaster relief camp might become something more permanent. To this end, SV collection in the camp will continue. It is likely that due to the limited disease surveillance in the camp the number of suspected and therefore laboratory-confirmed cases are an undercount. The confirmed cholera cases were marked manually on the map by the field team, but a more systematic digital recording, possibly using a web-based visualization system currently being developed for Goma, DRC, could locate cases in a more time-appropriate and accurate method, and even suggest where reporting deficiencies might exist. At least now the camp has a base map, and a system for updating risk, that can support the response to any future outbreak. At the same time, the machine learning work developed in Haiti is now being modified for these types of camps, with one of the main features being the automatic identification and mapping of tents (Figure 1E). The hope is that the techniques described in this paper are easily transferable to similar situations. While the length of SV collection varied with each month, the walking transects never took more than 50 min meaning that the on-the-ground logistical effort is minimal. If automatic image recognition and mapping can be applied, then making this an option for any new settlement mapping is plausible.

One topic that must also be considered in any challenging environment when applying field-based geospatial approaches is how these technologies will be received by the local population, especially those that are displaced. While the technologies involved, especially the body cameras, are small and unobtrusive, there have been previous SIS environments where security-caused caution has affected data collection. Similarly, there have been encounters where residents have challenged what the team were doing. However, in all these situations working with collaborators from the area, and who ideally live in or near the camp/SIS, has diffused tensions with a clear description of what is intended. It is vital that local collaborators are fully aware of any culturally specific situations that might cause concern in the area being served. It is also important to adhere to the typical types of cartographic and display privacy-preserving actions normally applied in more stable environments. Given the nature of SV data collection this includes masking any faces captured in images subsequently used for publications and presentations.

Future work into how the SV can be applied in other relief camps should be based around the transferability to different environments. For example, how frequently should transects be collected? How would local cultural and political conditions affect data collection? How might the completed maps be successfully used by local public health, and how can they be improved? To what degree is there inter-rater reliability issues in the manual coding of SV risks, and is it possible to standardize these in different environments? Are the current machine recognition strategies being developed for Mujoga appropriate for other camps in the DRC, or in other countries? All these next steps have already been considered with SV mapping of SIS environments. One common finding has been that every situation differs, and that flexibility in data collection, coding, analysis and mapping is always needed to maximize a successful outcome.

## 5. Conclusions

While the Mujoga relief camp has so far experienced only scattered cholera cases, regionally more extensive outbreaks had been recorded, meaning there is an ongoing concern for disease introduction. Many of the basic services and infrastructures initially bestowed on the camp have now gone, leaving a setting arguably more vulnerable to disease outbreaks than many SIS environments. Without SV mapping this camp would have remained a data-poor environment with little to no understanding of how life, and risk, was changing. Based on the example provided here, SV, enriched with context and supported by various data science advances in programming, display, and analysis is a viable option as a mapping approach for challenging environments. Even given the expected local variations that will be encountered, the flexibility of the various SV and SVG tools, including cameras and software, increases its suitability for adoption. Developing an international resource where equipment, software and expertise could be dispatched to any need as it arises would obviously improve the likelihood of implementation.

## Data Availability

Not applicable.

## References

[1] Sahasranaman A., Jensen H.J. (2021). Spread of COVID-19 in urban neighbourhoods and slums of the developing world. J. R. Soc. Interface.

[2] Abdi S., Wadugodapitiya A., Bedaf S., George C.E., Norman G., Hawley M., de Witte L. (2018). Identification of priority health conditions for field-based screening in urban slums in Bangalore, India. BMC Public Health.

[3] Sclar E.D., Garau P., Carolini G. (2005). The 21st century health challenge of slums and cities. Lancet.

[4] Shabane I., Nkambwe M., Chanda R. (2011). Landuse, policy, and squatter settlements: The case of peri-urban areas in Botswana. Appl. Geogr..

[5] Ezeh A., Oyebode O., Satterthwaite D., Chen Y.-F., Ndugwa R., Sartori J., Mberu B., Melendez-Torres G.J., Haregu T., Watson S.I. (2017). The history, geography, and sociology of slums and the health problems of people who live in slums. Lancet.

[6] Tjia D., Coetzee S. (2022). Geospatial information needs for informal settlement upgrading—A review. Habitat Int..

[7] Hachmann S., Arsanjani J.J., Vaz E. (2018). Spatial data for slum upgrading: Volunteered Geographic Information and the role of citizen science. Habitat Int..

[8] Berendes D.M., de Mondesert L., Kirby A.E., Yakubu H., Adomako L., Michiel J., Raj S., Robb K., Wang Y., Doe B. (2020). Variation in E. coli concentrations in open drains across neighborhoods in Accra, Ghana: The influence of onsite sanitation coverage and interconnectedness of urban environments. Int. J. Hyg. Environ. Health.

[9] Kraff N.J., Taubenböck H., Wurm M. How dynamic are slums? EO-based assessment of Kibera’s morphologic transformation. Proceedings of the 2019 Joint Urban Remote Sensing Event (JURSE).

[10] Brito P.L., Kuffer M., Koeva M., Pedrassoli J.C., Wang J., Costa F., Freitas A.D.d. (2020). The spatial dimension of COVID-19: The potential of earth observation data in support of slum communities with evidence from Brazil. ISPRS Int. J. Geo-Inf..

[11] Mahabir R., Croitoru A., Crooks A.T., Agouris P., Stefanidis A. (2018). A critical review of high and very high-resolution remote sensing approaches for detecting and mapping slums: Trends, challenges and emerging opportunities. Urban Sci..

[12] Sliuzas R., Kuffer M., Gevaert C., Persello C., Pfeffer K. Slum mapping. Proceedings of the 2017 Joint Urban Remote Sensing Event (JURSE).

[13] Lundine J., Kovačič P., Poggiali L. (2012). Youth and digital mapping in urban informal settlements: Lessons learned from participatory mapping processes in Mathare in Nairobi, Kenya. Child. Youth Environ..

[14] Falco E., Zambrano-Verratti J., Kleinhans R. (2019). Web-based participatory mapping in informal settlements: The slums of Caracas, Venezuela. Habitat Int..

[15] Mahabir R., Agouris P., Stefanidis A., Croitoru A., Crooks A.T. (2020). Detecting and mapping slums using open data: A case study in Kenya. Int. J. Digit. Earth.

[16] Ibrahim M.R., Haworth J., Cheng T. (2021). URBAN-i: From urban scenes to mapping slums, transport modes, and pedestrians in cities using deep learning and computer vision. Environ. Plan B Urban Anal. City Sci..

[17] Yeboah G., Porto de Albuquerque J., Troilo R., Tregonning G., Perera S., Ahmed S.A.S., Ajisola M., Alam O., Aujla N., Azam S.I. (2021). Analysis of openstreetmap data quality at different stages of a participatory mapping process: Evidence from slums in Africa and Asia. ISPRS Int. J. Geo-Inf..

[18] Kwan M.-P. (2016). Geographies of Health, Disease and Well-Being: Recent Advances in Theory and Method.

[19] Shannon K., Hast M., Azman A.S., Legros D., McKay H., Lessler J. (2019). Cholera prevention and control in refugee settings: Successes and continued challenges. PLoS Negl. Trop. Dis..

[20] Golicha Q., Shetty S., Nasiblov O., Hussein A., Wainaina E., Obonyo M., Macharia D., Musyoka R.N., Abdille H., Ope M. (2018). Cholera Outbreak in Dadaab Refugee Camp, Kenya—November 2015–June 2016. Morb. Mortal. Wkly. Rep..

[21] Mahamud A.S., Ahmed J.A., Nyoka R., Auko E., Kahi V., Ndirangu J., Nguhi M., Burton J.W., Muhindo B.Z., Breiman R.F. (2012). Epidemic cholera in Kakuma Refugee Camp, Kenya, 2009: The importance of sanitation and soap. J. Infect. Dev. Ctries..

[22] Bempah S., Curtis A., Awandare G., Ajayakumar J., Nyakoe N. (2022). The health-trash nexus in challenging environments: A spatial mixed methods analysis of Accra, Ghana. Appl. Geogr..

[23] Bempah S., Curtis A., Awandare G., Ajayakumar J. (2020). Appreciating the complexity of localized malaria risk in Ghana: Spatial data challenges and solutions. Health Place.

[24] Curtis A., Squires R., Rouzier V., Pape J.W., Ajayakumar J., Bempah S., Taifur Alam M., Alam M.M., Rashid M.H., Ali A. (2019). Micro-Space Complexity and Context in the Space-Time Variation in Enteric Disease Risk for Three Informal Settlements of Port au Prince, Haiti. Int. J. Environ. Res. Public Health.

[25] Mills J.W., Curtis A., Kennedy B., Kennedy S.W., Edwards J.D. (2010). Geospatial video for field data collection. Appl. Geogr..

[26] Curtis A.J., Mills J.W., McCarthy T., Fotheringham A.S., Fagan W.F. (2009). Space and time changes in neighborhood recovery after a disaster using a spatial video acquisition system. Geospatial Techniques in Urban Hazard and Disaster Analysis.

[27] Curtis A., Mills J.W., Kennedy B., Fotheringham S., McCarthy T. (2007). Understanding the geography of Post-Traumatic stress: An academic justification for using a spatial video acquisition system in the response to hurricane katrina. J. Contingencies Crisis Manag..

[28] Curtis A., Fagan W.F. (2013). Capturing damage assessment with a spatial video: An example of a building and street-scale analysis of tornado-related mortality in Joplin, Missouri 2011. Ann. Assoc. Am. Geogr..

[29] Curtis A., Mills J.W. (2012). Spatial video data collection in a post-disaster landscape: The Tuscaloosa Tornado of April 27th 2011. Appl. Geogr..

[30] Curtis A., Blackburn J.K., Smiley S.L., Yen M., Camilli A., Alam M.T., Ali A., Morris J.G. (2016). Mapping to support fine scale epidemiological cholera investigations: A case study of spatial video in Haiti. Int. J. Environ. Res. Public Health.

[31] Curtis A., Blackburn J.K., Widmer J.M., Morris Jr J.G. (2013). A ubiquitous method for street scale spatial data collection and analysis in challenging urban environments: Mapping health risks using spatial video in Haiti. Int. J. Health Geogr..

[32] Curtis A., Quinn M., Obenauer J., Renk B.M. (2017). Supporting local health decision making with spatial video: Dengue, Chikungunya and Zika risks in a data poor, informal community in Nicaragua. Appl. Geogr..

[33] Smiley S.L., Curtis A., Kiwango J.P. (2017). Using spatial video to analyze and map the water-fetching path in challenging environments: A case study of Dar es Salaam, Tanzania. Trop. Med. Infect. Dis..

[34] Bempah S., Odhiambo L., Curtis A., Pandit A., Mofleh D., Ajayakumar J., Odhiambo L.A. (2021). Fine Scale Replicable Risk Mapping in an Informal Settlement: A Case Study of Mathare, Nairobi. J. Health Care Poor Underserved.

[35] Curtis A., Bempah S., Ajayakumar J., Mofleh D., Odhiambo L. (2018). Spatial Video Health Risk Mapping in Informal Settlements: Correcting GPS Error. Int. J. Environ. Res. Public Health.

[36] Krystosik A.R., Curtis A., Buritica P., Ajayakumar J., Squires R., Dávalos D., Pacheco R., Bhatta M.P., James M.A. (2017). Community context and sub-neighborhood scale detail to explain dengue, chikungunya and Zika patterns in Cali, Colombia. PLoS ONE.

[37] Ajayakumar J., Curtis A.J., Rouzier V., Pape J.W., Bempah S., Alam M.T., Alam M.M., Rashid M.H., Ali A., Morris J.G. (2022). Spatial Video and EpiExplorer: A Field Strategy to Contextualize Enteric Disease Risk in Slum Environments. Int. J. Environ. Res. Public Health.

[38] Curtis A., Curtis J.W., Shook E., Smith S., Jefferis E., Porter L., Schuch L., Felix C., Kerndt P.R. (2015). Spatial video geonarratives and health: Case studies in post-disaster recovery, crime, mosquito control and tuberculosis in the homeless. Int. J. Health Geogr..

[39] Krystosik A.R., Curtis A., LaBeaud A.D., Dávalos D.M., Pacheco R., Buritica P., Álvarez Á.A., Bhatta M.P., Rojas Palacios J.H., James M.A. (2018). Neighborhood violence impacts disease control and surveillance: Case study of Cali, Colombia from 2014 to 2016. Int. J. Environ. Res. Public Health.

[40] Ajayakumar J., Curtis A., Smith S., Curtis J. (2019). The use of geonarratives to add context to fine scale geospatial research. Int. J. Environ. Res. Public Health.

[41] Ma C., Zhao Y., Curtis A., Kamw F., Shamal A.-D., Yang J., Jamonnak S., Ali I. (2020). CLEVis: A Semantic Driven Visual Analytics System for Community Level Events. IEEE Comput. Graph. Appl..

[42] Jamonnak S., Zhao Y., Curtis A., Al-Dohuki S., Ye X., Kamw F., Yang J. (2020). GeoVisuals: A visual analytics approach to leverage the potential of spatial videos and associated geonarratives. Int. J. Geogr. Inf. Sci..

[43] Tyner J.A., Curtis A., Kimsroy S., Chhay C. (2018). The evacuation of phnom penh during the cambodian genocide: Applying spatial video geonarratives to the study of genocide. Genocide Stud. Prev. Int. J..

[44] Curtis A., Tyner J., Ajayakumar J., Kimsroy S., Ly K.-C. (2019). Adding spatial context to the april 17, 1975 evacuation of phnom penh: How spatial video geonarratives can geographically enrich genocide testimony. GeoHumanities.

[45] Jamonnak S., Bhati D., Amiruzzaman M., Zhao Y., Ye X., Curtis A. (2022). Visual Community: A platform for archiving and studying communities. J. Comput. Soc. Sci..

[46] Ajayakumar J., Curtis A.J., Rouzier V., Pape J.W., Bempah S., Alam M.T., Alam M.M., Rashid M.H., Ali A., Morris J.G. (2021). Exploring convolutional neural networks and spatial video for on-the-ground mapping in informal settlements. Int. J. Health Geogr..

[47] Manirambona E., Uwiringiyimana E., Musa S.S., Niyonkuru S., Gyeltshen D., Adebisi Y.A., Lucero-Prisno D.E. (2022). Impact of Nyiragongo Volcanic Eruptions on the Resilience to the COVID-19 and Ebola in the Democratic Republic of the Congo. Ann. Public Health.

[48] Dorsainvil M. (2021). Cholera: Still a Major Public Health Issue in Sub-Saharan Africa. J. Health Care Poor Underserved.

